# Psychological Problems and Socioemotional Wellbeing among Children of Mothers with Depression and Their Association with Sociodemographic Factors in a Sri Lankan Setting

**DOI:** 10.1155/2018/3809384

**Published:** 2018-04-16

**Authors:** Yasodha Maheshi Rohanachandra, Shamini Prathapan, Swarna Wijetunge

**Affiliations:** ^1^Department of Psychiatry, Faculty of Medical Sciences, University of Sri Jayewardenepura, Nugegoda, Sri Lanka; ^2^Department of Community Medicine, Faculty of Medical Sciences, University of Sri Jayewardenepura, Nugegoda, Sri Lanka; ^3^Lady Ridgeway Hospital for Children, Colombo 08, Sri Lanka

## Abstract

**Background:**

Maternal depression is known to be associated with anxiety, depression, oppositional and conduct disorders, neurocognitive deficits, ADHD, substance abuse, and personality disorder in the offspring. We aimed to describe the proportion of psychological problems among children of mothers with depression in Sri Lanka and to describe the associated sociodemographic factors.

**Methods:**

A cross-sectional descriptive study was conducted on 100 children and adolescents between 4 and 16 years, whose mothers have a diagnosis of depression and are currently in remission. Specifically designed instruments were used to extract sociodemographic details and data on mother's illness. Strengths and Difficulties Questionnaire (SDQ) was used to detect psychological problems in children.

**Results:**

14% of the children scored abnormally high in hyperactivity, 13% in conduct problems, 12% in emotional problems, 9% in peer problems, and 10% in total difficulties. Children (4–12 years) scored significantly higher than the adolescents in hyperactivity and externalizing problems (*p* < 0.05). Significant differences were also found in peer problems (*p* < 0.05), internalizing problems (*p* < 0.05), and total difficulties (*p* < 0.05) in relation to the age of the mother.

**Conclusion:**

Screening the children of mothers with depression for psychological problems and developing a holistic management plan, which includes measures to ensure their wellbeing, is important.

## 1. Introduction

Maternal depression, occurring either postpartum or later in life, has been shown to be associated with many adverse health outcomes among the offspring. Early reviews revealed some 40–45% of children of parents with affective disorders to be suffering from psychiatric disorders [1], with later studies showing a lower prevalence of 18% [2]. It has been shown that the incidence of psychopathology in children of depressed mothers is 3-4 times higher than in children of nondepressed mothers [3]. The offspring of depressed mothers have been shown to have higher rates of developmental delays, somatic complaints, sleep problems, child abuse, and psychiatric and neurobehavioural disorders such as depression, anxiety disorders, symptoms of Attention Deficit Hyperactivity Disorder (ADHD), conduct and oppositional disorders, language and cognitive delays, and decreased perceptual performance compared to the children of nondepressed mothers [4–11]. Previous studies have also shown that the infants of depressed mothers have an increased risk of avoidant and disorganized attachment. Antisocial behavior in adolescents has also been noted to be associated with maternal depression [12]. Poor physical health during early childhood, resulting in increased health related stress in the young adult offspring of depressed mothers, has also been reported [13].

Several factors determine the effect of maternal depression on child's emotional health. Previous studies have demonstrated that, in infancy and early childhood, maternal postnatal depression has a greater impact on boys than girls [12]. Studies on the adolescent offspring of depressed mothers have shown a higher risk of behavioural problems in adolescent boys and a higher risk of depression and internalizing problems in adolescent girls [14, 15]. Some previous studies have demonstrated that the impact on children in families where the mother is depressed is linked to the number of children and in families with more than 2 siblings having a higher risk of language delays compared to families with less number of children [12]. However, despite the high proportion of psychological problems described in children, some children show resilience to the effects of their mothers' depression and cope effectively. Factors that are known to mitigate the effects of maternal depression on the offspring include family factors such as close relationship with parents/guardian, authoritative parenting, being connected to an extended family, and especially having a good relationship with grandparents. Child factors such as good intellectual functioning, easy temperament, high self-esteem, and factors outside the family such as good schooling and satisfactory relationships with other adults also act as buffers [16].

Much of the literature on effects of maternal depression on children comes from studies conducted in Western countries with only a few studies being done in Asian countries. The impact of maternal depression on children has not yet been studied in Sri Lanka. This study will be useful in identifying the magnitude of this problem in Sri Lanka and in assessing the contributing sociodemographic factors, which will aid in identifying children at risk, designing guidelines for assessment of affected children, and planning preventive strategies to minimize the impact of maternal depression on their children in Sri Lanka.

## 2. Materials and Methods

### 2.1. Study Design and Setting

This is a quantitative research with a descriptive cross-sectional design. The study was conducted at the outpatient general adult psychiatry follow-up clinics of Colombo South Teaching Hospital and The National Hospital of Sri Lanka.

### 2.2. Study Population

The study sample included female patients who have been diagnosed by the Consultant Psychiatrist to have either a depressive episode (mild, moderate, severe, or severe with psychotic symptoms) or recurrent depressive disorder in accordance with International Classification of Diseases 10th edition (ICD 10) [17], who are currently in remission and having children aged between 4 and 16 years. Patients who have been diagnosed as having bipolar depression, patients who are not the primary caregiver for their children, and patients whose children are having intellectual disability were excluded from the study. When the mother has more than one child between 4 and 16 years, only the first child was included in the study.

### 2.3. Sample Size and Sampling Technique

A sample size of 100 was calculated using a confidence level of 95%, a margin of error of 7.5%, and an estimated prevalence of 18% according to the previous literature [2]. The first 100 patients who fulfilled the inclusion and exclusion criteria were included in the study.

### 2.4. Study Instruments and Data Collection

A specifically designed self-administered questionnaire was used to collect data on sociodemographic factors. The Peradeniya Depression Scale (PDS) was used to assess and to determine the presence of depressive symptoms in the study population, in order to identify those who are currently in remission. The PDS is a screening tool for depression which was developed in Sri Lanka and validated among outpatients presenting to a psychiatry clinic in a government hospital in Peradeniya [18]. PDS consists of 25 items under 5 categories. It is designed to look for the presence or absence of depressive symptoms over the preceding 2 weeks and has a sensitivity of 88.5% and specificity of 85%. PDS is demonstrated to be a highly accurate tool for the detection of depression in Sri Lanka [18].

The parent rated version of the Strengths and Difficulties Questionnaire (SDQ) was used to assess the presence of psychological problems among the children [19]. The SDQ has previously been translated to Sinhala and validated in Sri Lanka [19]. The SDQ has 5 subscales with 5 items each. The 5 subscales include emotional problems, hyperactivity, conduct problems, peer problems, and prosocial behaviour.

A structured clinical interview based on ICD research criteria was arranged to assess for the presence of a psychiatric disorder in children with borderline or abnormal scores in any of the subscales of the Strengths and Difficulties Questionnaire (SDQ).

### 2.5. Data Collection and Analysis

A pilot study was initially carried out on a sample of 10 patients prior to the actual study, to test the feasibility of the study and to detect any practical difficulties. Data were summarized and analyzed using the Statistical Package for the Social Sciences (SPSS 22.0). Scores for hyperactivity, conduct problems, emotional problems, peer problems, externalizing problems (sum of hyperactivity and conduct problems), internalizing problems (sum of emotional problems and peer problems), and a total difficulties score were calculated. The independent samples *t*-test was used in examining the association between SDQ scores of the two groups and one-way ANOVA was used in examining the association between SDQ scores of more than two groups. Associations that generated a *p* value of less than 0.05 were considered as true associations.

### 2.6. Ethical Considerations

Ethical approval was obtained from the ethical review committee of the Colombo South Teaching Hospital. Informed consent was obtained from all participants.

## 3. Results

### 3.1. Sociodemographic Characteristics

The mean age of the children assessed was 10.73 years. 60% (*n* = 60) of the children were between 4 and 12 years, while 40% (*n* = 40) of the children were between 12 and 16 years. 59% (*n* = 59) of the children were males. The age of the mother ranged from 25 to 58 years, with a mean age of 39.19 years (Table 1).

### 3.2. Psychological Problems in Children

Ten percent (*n* = 10) of the children scored abnormally high in total difficulties score. 2% (*n* = 2) had abnormally low scores on prosocial behaviour (Table 2).

4% (*n* = 4) of the children had an ICD-10 diagnosis. 2% (*n* = 2) of the children had Attention Deficit Hyperactivity Disorder, 1% (*n* = 1) had Attention Deficit Hyperactivity Disorder with Comorbid Conduct Disorder, and further 1% (*n* = 1) had Depression with Comorbid Obsessive Compulsive Disorder.

### 3.3. Factors Associated with Psychological Problems in Children

There were statistically significant differences in peer problems (*F* = 6.53, *p* ≤ 0.001), internalizing problems (*F* = 7.00, *p* ≤ 0.001), and total difficulties (*F* = 4.14, *p* = 0.008) among the children studied, in relation to the age of the mother. Children of mothers aged more than 50 years showed higher score in peer problems (*M* = 3.50, SD = 3.78) compared to children of mothers aged between 30 and 40 years (*M* = 0.78, SD = 1.25) and 40 and 50 years (*M* = 1.03, SD = 1.26). Children whose mothers were between 30 and 40 years had significantly lower internalizing problems (*M* = 2.22, SD = 2.41) than children whose mothers were either between 20 and 30 years (*M* = 5.36, SD = 3.23) or more than 50 years (*M* = 6.50, SD = 5.01). Total difficulties score varied significantly between children of mothers aged 20–30 years (*M* = 12.0, SD = 5.53) and 30–40 years (*M* = 6.56, SD = 5.13), with children of mothers aged between 20 and 30 years having a significantly higher total difficulties score than children of mothers aged between 30 and 40 years.

The test revealed a statistically significant difference between children (4–12 years) and adolescents (12–16 years) in the hyperactivity score (*t* = 3.02, df = 96.48, *p* = 0.003), with children (*M* = 4.07, SD = 3.09) scoring significantly higher in the hyperactivity score than the adolescents (*M* = 2.43, SD = 2.33). In addition, there was a statistically significant difference between children (4–12 years) and adolescents (12–16 years) in the score for externalizing problems (*t* = 2.63, df = 97.52, *p* = 0.01), where children (*M* = 5.80, SD = 4.50) had a significantly higher score than the adolescents (*M* = 3.78, SD = 3.20).

There was no statistically significant difference in psychological problems in relation to the mothers' education level, mothers' occupational status, number of children in the family, gender of the child, or the current living circumstance.

## 4. Discussion

The present study revealed abnormally high total emotional and behavioural difficulties score in 10% of the children studied, with 2% of the children having abnormally low scores on prosocial behaviour. This is comparable to the findings of a follow-up study done in Pakistan, which found 13.3% of the children of mothers with depression to have an abnormal total difficulties score at 7 years [20]. However, the rates of conduct problems were much higher in the study conducted in Pakistan (47.1% of boys; 34.7% of girls) than in the current study where conduct problems were present in 13.1% of children. The difference in the rate of conduct problems may be explained by the difference in the age group in the sample. The previous study assessed children at 7 years of age while 13% of our sample consisted of children less than 7 years. Some of the questions measuring conduct problems, such as lying/deceiving and stealing, may not manifest in children less than 7 years, which may be the reason for conduct problems being lower in the present study.

Many studies have shown rates of psychological problems in children of depressed mothers to be higher than in the children of nondepressed mothers [4–11]. A follow-up study in Pakistan showed that the SDQ scores were highest in children whose mothers were depressed both perinatally and currently and decreased in a stepwise pattern for children whose mothers were only depressed currently, mothers who were only depressed perinatally, to mothers who were never depressed [20]. Similar patterns have been demonstrated in a study done in Australia, where 23.9% of children whose mothers have persistent and increasing depressive symptoms have SDQ scores in the clinical range, with proportions decreasing to 19.1% when there are subclinical depressive symptoms and to 7% when there are minimal depressive symptoms [21]. In the present study, we did not compare the proportion of psychological problems in children without a history of maternal depression.

The present study revealed a higher proportion of hyperactivity and externalizing problems with children when compared to adolescents. This finding is supported by a meta-analysis by Goodman et al. [15], which revealed that associations between depression in mothers and children's internalizing and externalizing problems and general psychopathological issues are stronger for younger children than older children and adolescents. Reasons that have been postulated to explain this difference include the following: (a) children who had their first exposure to maternal depression at an older age may have experienced more years of normal development before being exposed to the adverse effects of their mothers depression; (b) as children grow older, they become less dependent on the mother and develop a wider network of relationships including the father, teachers, and peers which may help mitigate the effects of the maternal depression; (c) as children become more cognitively mature, they may be able to attribute the mothers' behaviour as an illness compared to younger children and they may also have developed better emotion regulation and social information processing skills [22, 23].

The findings showed that children whose mothers were less than 30 years or more than 50 years had more emotional and behavioural problems than children of mothers between 30 and 50 years. We could not find any studies which examined the age of the mother as a modulator of risk in the development of psychological problems in children where the mother is depressed. However, it is possible to postulate that mothers who are older than 50 years may have less energy and possibly have more physical comorbidities than younger mothers between 30 and 50 years. Therefore, these mothers may find it even harder than younger mothers to care for their children in the presence of depression due to the premorbid difficulties with lack of energy and physical comorbidities. It is also possible to postulate that mothers who are younger than 30 years may have less maturity and suboptimal coping skills than mothers of 30–50 years, which may make it harder for these mothers to cope with demands of rearing children in the presence of depression.

Some previous studies have demonstrated that, in families where the mother is depressed, the number of children in the family is linked to the adverse outcome; families with 2 or more children were shown to have a higher risk of language delays compared to families with less than 2 children. This was postulated to be due to the mother being less available to meet the demands of the child in a larger family [24]. This finding was not consistent with the results of the present study as we did not find an association between the number of children in the family and the psychological problems in children. This difference may be explained by the difference in the composition of the study sample. Sixteen percent of the mothers in the previous study were not living with their partner, whereas 97% of the mothers in our study were married and living with their husbands. Having the support of a partner in raising children may have mitigated the adverse effects of having a large family, which may explain our findings of the psychological problems having no association with the number of children in the family.

Screening for depression in pregnancy and postpartum period as a part of routine medical investigation has been recommended by previous studies [25–27] and has been shown to be effective in detecting high risk women. However, due to lack of resources, screening for depression either during pregnancy or postpartum period does not take place in Sri Lanka at present. In addition, although the existing evidence suggests that family based interventions [27], parenting programmes, and home visiting programmes [28] improve social and emotional problems in the offspring of mothers with depression, currently no such practice exists in Sri Lanka. Treatment of maternal depression, especially occurring beyond the postpartum period, focuses mainly on the mother and is treated with psychotropic medications and standard cognitive behavioural therapy. Due to the availability of limited resources in Sri Lanka, the impact of maternal depression on the psychological wellbeing on children is often overlooked. As this study reveals a high proportion of children to have psychological difficulties, it highlights the need to screen for depressive symptoms in pregnancy and to develop an alternative, more family focused approach to the treatment of maternal depression in Sri Lanka.

## 5. Limitations

This study was carried out in follow-up clinics at The National Hospital of Sri Lanka and Colombo South Teaching Hospital, which mainly cater to patients from the Colombo district. Therefore, the results may not be applicable to the entire Sri Lankan population. Previous literature has shown that the association between maternal depression and child outcomes is likely to be stronger when the depressed mother was the source of information to the child, relative to other sources or to the child's self-report, especially when the mother has a high degree of depressive symptoms [15]. This may be considered as a limitation of our study as we gathered information only from the mother. However, we tried to minimize this error by including only mothers who are currently in remission. This study did not compare the magnitude of psychological problems in children of depressed mothers to nondepressed mothers, which is another limitation of this study.

## 6. Conclusions

Ten percent of children whose mothers had a depressive disorder had emotional or behavioural problems. Sociodemographic factors associated with higher psychological problems in children were the younger age of the child and age of the mother (either less than 30 years or more than 50 years). As a substantial proportion of children had psychological problems, screening for emotional and behavioural problems in children where mother is diagnosed to have depression, especially if children are younger or if the mother is young (less than 30 years) or elderly (more than 50 years), is important in early identification of problems. When developing a management plan for the mother with depression, special considerations should be given to include steps to minimize the impact of the illness on the child. Education of health professionals about the detrimental effects of maternal depression on the child's mental health would also aid in the early identification of problems in children and is important in reducing the impact of maternal depression on the child.

## Figures and Tables

**Table 1:** Sociodemographic characteristics (study sample: *N* = 100 and the age: 4–16 years).

Sociodemographic variables	Frequency	Percent
Age of the child		
4–≤12 years	60	60
12–≤16 years	40	40
Sex of the child		
Male	59	59
Female	41	41
Mother's age		
20–30	11	11
30–40	50	50
40–50	33	33
>50	06	06
Mother's education level		
No school education	04	04
Years 1–5	02	02
Years 6–11	56	56
Passed O/L	16	16
Years 12-13	10	10
Passed A/L	11	11
Graduate	01	01
Mother's employment status		
Employed	76	76
Not employed	24	24
Mother's marital status		
Married	88	88
Separated	11	11
Widowed	01	01
Number of children in the family		
1	19	19
2	32	32
3	35	35
4	06	06
5	08	08
Birth order of the child		
First	21	21
Second	37	37
Third	15	15
Fourth	06	06
Fifth	03	03
Only child	18	18
Living circumstances		
Nuclear family	64	64
Extended family	36	36

**Table 2:** Proportion of psychological problems in children.

Domain	Average (%)	Borderline (%)	Abnormal (%)	Missing (%)
Hyperactivity	76	09	14	01
Conduct problems	76	11	13	-
Emotional problems	81	06	12	01
Peer problems	87	04	09	-
Prosocial behavior	96	02	02	-
Total difficulties	85	05	10	-

## References

[1] Beardslee W. R., Bemporad J., Keller M. B., Klerman G. L. (1983). Children of parents with major affective disorders: a review. *The American Journal of Psychiatry*.

[2] Luoma I., Tamminen T., Kaukonen P. (2001). Longitudinal study of maternal depressive symptoms and child well-being. *Journal of the American Academy of Child and Adolescent Psychiatry*.

[3] Cummings E. M., Davies P. T., Joiner T., Coyne J. C. (1999). Depressed parents and family functioning: interpersonal effects and children’s functioning and development. *The Interactional Nature of Depression: Advances in Interpersonal Approaches*.

[4] Beardslee W. R., Versage E. M., Gladstone T. R. G. (1998). Children of affectively ill parents: a review of the Past 10 Years. *Journal of the American Academy of Child & Adolescent Psychiatry*.

[5] Weissman M. M., Warner V., Wickramaratne P., Moreau D., Olfson M. (1997). Offspring of depressed parents: 10 years later. *Archives of General Psychiatry*.

[6] Chronis A. M., Pelham W. E., Baumann B. L. (2007). Maternal depression and early positive parenting predict future conduct problems in young children with attention-deficit/hyperactivity disorder. *Developmental Psychology*.

[7] Quevedo L. A., Silva R. A., Godoy R. (2012). The impact of maternal post-partum depression on the language development of children at 12 months. *Child: Care, Health and Development*.

[8] Murray L., Fiori-Cowley A., Hooper R., Cooper P. (1996). The impact of postnatal depression and associated adversity on early mother-infant interactions and later outcomes. *Child Development*.

[9] Martini J., Petzoldt J., Knappe S., Garthus-Niegel S., Asselmann E., Wittchen H.-U. (2017). Infant, maternal, and familial predictors and correlates of regulatory problems in early infancy: The differential role of infant temperament and maternal anxiety and depression. *Early Human Development*.

[10] Koutra K., Roumeliotaki T., Kyriklaki A. (2017). Maternal depression and personality traits in association with child neuropsychological and behavioral development in preschool years: Mother-child cohort (Rhea Study) in Crete, Greece. *Journal of Affective Disorders*.

[11] Antúnez Z., de la Osa N., Granero R., Ezpeleta L. (2018). Reciprocity Between Parental Psychopathology and Oppositional Symptoms From Preschool to Middle Childhood. *Journal of Clinical Psychology*.

[12] Shaw D. S., Hyde L. W., Brennan L. M. (2012). Early predictors of boys' antisocial trajectories. *Development and Psychopathology*.

[13] Raposa E., Hammen C., Brennan P., Najman J. (2014). The long-term effects of maternal depression: Early childhood physical health as a pathway to offspring depression. *Journal of Adolescent Health*.

[14] Rutter M. (1990). Commentary: Some Focus and Process Considerations Regarding Effects of Parental Depression on Children. *Developmental Psychology*.

[15] Goodman S. H., Rouse M. H., Connell A. M., Broth M. R., Hall C. M., Heyward D. (2011). Maternal Depression and Child Psychopathology: A Meta-Analytic Review. *Clinical Child and Family Psychology Review*.

[16] Williams H., Carmichael A. (1985). Depression in mothers in a multi‐ethnic urban industrial municipality in melbourne. aetiological factors and effects on infants and preschool children. *Journal of Child Psychology and Psychiatry*.

[17] ICD-10 Classifications of Mental and Behavioural Disorder: Clinical Descriptions and Diagnostic Guidelines. Geneva. World Health Organization. 1992

[18] Abeyasinghe D. R. R., Tennakoon S., Rajapakse T. N. (2012). The development and validation of the Peradeniya Depression Scale (PDS) - A culturally relevant tool for screening of depression in Sri Lanka. *Journal of Affective Disorders*.

[19] Perera S., Thalagala E., Chandrarathna S. H., Agampodi T. C., Nugegoda D. B., Agampodi S. B. (2013). Factor structure and normative data of the Sinhalese version of self reported Strength and Difficulties Questionnaire (SDQ) for adolescents. *The Ceylon Medical Journal*.

[20] Maselko J., Sikander S., Bangash O. (2016). Child mental health and maternal depression history in Pakistan. *Social Psychiatry and Psychiatric Epidemiology*.

[21] Giallo R., Woolhouse H., Gartland D., Hiscock H., Brown S. (2015). The emotional–behavioural functioning of children exposed to maternal depressive symptoms across pregnancy and early childhood: a prospective Australian pregnancy cohort study. *European Child and Adolescent Psychiatry*.

[22] Crick N. R., Dodge K. A. (1996). Social Information-Processing Mechanisms in Reactive and Proactive Aggression. *Child Development*.

[23] Grych J. H., Fincham F. D. (1990). Marital conflict and children's adjustment: A cognitive-contextual framework. *Psychological Bulletin*.

[24] Halpern R., Giugliani E. R., Victora C. G., Barros C. B., Horta B. L. (2000). Risk factors for suspicion of development delays at 12 months of age. *Journal of Pediatrics*.

[25] O'Connor E., Rossom R. C., Henninger M., Groom H. C., Burda B. U. (2016). Primary care screening for and treatment of depression in pregnant and postpartumwomen evidence report and systematic review for the US preventive services task force. *Journal of the American Medical Association*.

[26] Walfisch A., Sermer C., Matok I., Koren G., Einarson A. (2011). Screening for depressive symptoms. *Canadian Family Physician*.

[27] Hanusa B. H., Scholle S. H., Haskett R. F., Spadaro K., Wisner K. L. (2008). Screening for depression in the postpartum period: a comparison of three instruments. *Journal of Women's Health*.

[28] Goodman S. H., Garber J. (2017). Evidence-based interventions for depressed mothers and their young children. *Child Development*.

